# Sudden Bilateral Hearing Loss Is a Neurovascular Emergency

**DOI:** 10.1155/crnm/5892582

**Published:** 2026-11-01

**Authors:** Muhammad Aemaz Ur Rehman, Mohammad Aladawi, Eroboghene E. Ubogu, Felix Guerra-Castanon

**Affiliations:** ^1^ Department of Neurology University of Alabama at Birmingham Birmingham Alabama USA uab.edu

## Abstract

Sudden bilateral hearing loss is not commonly encountered by neurologists. Many first‐line providers commonly label hearing loss as peripheral in origin, leading to misdiagnosis and delayed treatment. A 49‐year‐old female presented with sudden bilateral hearing loss and headache. Neurologic examination revealed mild right‐sided weakness, sensory loss, and ataxia. CT scan of the brain revealed left mid‐dorsal pontine intraparenchymal hemorrhage. Further testing with MRI brain and brainstem auditory evoked potentials (BAEPs) confirmed a central etiology of hearing loss. Prompt diagnosis and treatment resulted in complete resolution of hearing loss and only mild, nondisabling sensorimotor deficits at 3‐month clinic follow‐up. This case emphasizes the importance of treating sudden‐onset bilateral hearing loss as a neurologic emergency until proven otherwise. We discuss the etiologies of sudden‐onset sensorineural hearing loss and carefully localize the lesion to the brainstem in this rare presentation of a pontine syndrome. We highlight common principles of BAEP testing and expected findings in patients with stroke and demyelinating lesions.

## 1. Introduction

Sudden bilateral hearing loss is a rare clinical entity. It can be conductive (e.g., cerumen impaction), sensorineural (e.g., brainstem ischemia), or mixed. The estimated incidence of sudden‐onset sensorineural hearing loss (SSNHL) ranges from 5 to 20 per 100,000, with the vast majority being unilateral [1–3]. Bilateral SSNHL is a very rare condition comprising 0.44%–3.4% of the cases, but more importantly, it is an otologic emergency and has been associated with more severe hearing loss and poorer prognosis [1]. Etiologies for bilateral SSNHL include toxic, infectious, vascular, neoplastic, traumatic, autoimmune, and idiopathic [2]. Infective, toxic, and autoimmune etiologies are more likely in younger patients, whereas vascular and neoplastic causes are more likely in older populations [3]. Infective, neoplastic, and autoimmune etiologies are also typically more insidious in onset than vascular, which is typically hyperacute. Because the central auditory pathway decussates and ascends bilaterally through a compact region of the pons [4], a single central lesion can produce bilateral hearing loss, and life‐threatening vascular causes (e.g., basilar artery occlusion and brainstem hemorrhage) may present with bilateral hearing loss as an early or isolated symptom before other brainstem signs emerge [3].

## 2. Case Presentation

We present a 49‐year‐old female with hypertension (noncompliant with medications), congestive heart failure, and migraines who presented to a tertiary care hospital with sudden bilateral hearing loss. The patient had reported intermittent headaches 5 days before admission, which she reported felt like her typical migraines. Suddenly, she was woken up from her sleep with a pounding headache and hearing loss associated with mild tinnitus. There were no recently reported fevers, neck pain, ear pain, vertiginous episodes, ear drainage, upper respiratory tract infections, and facial or head trauma. On general physical examination, she appeared anxious with no evidence of face or head ecchymoses or laceration. Otologic exam showed normal bilateral tympanic membranes and auditory canals. She was afebrile with normal vital signs except for notably elevated blood pressure of 229/147 mmHg. Neurologic examination revealed normal mental status, left gaze palsy, decreased sensation in the right limbs, and mild right upper‐ and lower‐extremity pronator drift and ataxia. In addition, she could not repeat whispered numbers in either ear. Facial strength and sensation were intact, and no other cranial nerve deficits were appreciated. Basic laboratory workup, including complete blood count and complete metabolic panel, was unremarkable. However, the urine drug screen was positive for amphetamines and buprenorphine (illicit drug use). She was not on any ototoxic home medications and denied a family history of hearing loss.

The neurologic examination in this patient revealed a left horizontal gaze palsy accompanied by bilateral hearing loss as well as subtle right‐sided pronator drift, ataxia, and sensory impairment. This constellation of findings strongly suggests a central lesion which most likely localizes to the brainstem, specifically the pons, where the auditory and ocular motor systems anatomically converge. The horizontal gaze palsy implicates involvement of the paramedian pontine reticular formation (PPRF), which resides in the dorsal pons and is essential for ipsilateral horizontal (lateral conjugate) gaze [4].

To localize the bilateral hearing loss more precisely, it is important to understand the central auditory pathway. After synapsing in the cochlear nuclei at the pontomedullary junction, some auditory fibers decussate via the trapezoid body in the caudal pons and ascend bilaterally through the lateral lemnisci to the inferior colliculi [4]. The trapezoid body is the principal decussation point for auditory fibers, where fibers from both cochlear nuclei converge and cross within a compact midline structure. A unilateral hemorrhage centered in the dorsomedial pons can, therefore, disrupt crossing fibers originating from both sides, producing bilateral hearing loss even though the hemorrhage itself is lateralized. This mechanism was proposed by Egan et al. [5], who demonstrated that inactivation of the ventral acoustic striae decussating in the trapezoid body could account for bilateral total deafness from a predominantly midline pontine hemorrhage. In this case, the combination of bilateral hearing loss with horizontal gaze palsy suggests the involvement of the caudal pons affecting both the decussating auditory fibers (trapezoid body) and adjacent gaze control structures. The minor right‐sided drift, ataxia, and sensory changes further support a unilateral but extensive pontine process that is unlikely to be ischemic, given multiple artery beds that irrigate the different tracts. Taken together, these findings underscore the value of careful clinical localization and raise concern for a pontine lesion affecting multiple longitudinal systems within a compact anatomical area.

The first‐line tests in this patient should include a noncontrast CT (NCCT) of the head and a CT angiogram (CTA) of the head and neck. The primary purpose of the NCCT is to rapidly exclude acute intraparenchymal hemorrhage (IPH), while the CTA helps evaluate for arterial occlusions, vascular malformations, and blood extravasation. Given the acute bilateral hearing loss, new‐onset horizontal gaze palsy, elevated blood pressure, and headache, hemorrhagic stroke, particularly in the brainstem, should be our primary consideration. These symptoms may also raise suspicion for posterior circulation ischemia or, less commonly, central nervous system infection or autoimmune process. In this case, the initial NCCT showed hyperdensity in the left dorsal medial pons, representing an acute hemorrhage (Figure 1a–c). There was no extension into the 4th ventricle, no subarachnoid blood, and no significant mass effect or obstructive hydrocephalus. The absence of a spot sign on CTA suggested no active extravasation, and no underlying aneurysm, arteriovenous malformation, or dural arteriovenous fistula was identified. Blood pressure control and standard IPH management became the immediate priorities, including admission to the neurocritical care unit, serial neurologic examinations, surveillance imaging, and temporary discontinuation of antithrombotic medications.

**FIGURE 1:**
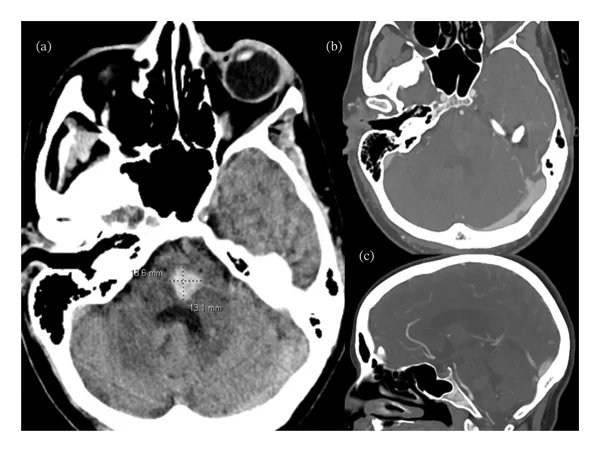
(1a) Initial noncontrast CT head showing 13.6 × 13.1‐mm hyperdensity indicating acute IPH. (1b,c) CT angiogram showing filling of the basilar artery at the level of the hemorrhage without any obvious vascular malformation and negative spot sign.

MRI of the brain with and without contrast was obtained after clinical stabilization to further characterize the lesion and rule out secondary causes such as cavernous malformation, neoplasm, or demyelination. MRI revealed a 1.4 × 1.3‐cm lesion with susceptibility artifact, hypointense on T2/FLAIR, and isointense on T1, with mild surrounding FLAIR hyperintensity, representing an acute dorsomedial pontine IPH extending into the left pons with mild perilesional edema. There was no diffusion restriction on diffusion‐weighted imaging (DWI), no contrast enhancement or obvious underlying mass lesion on postcontrast T1, and a 4‐month follow‐up MRI revealed expected hemorrhage evolution without underlying cavernous malformation. Additional neuroimaging findings, including Fazekas Grade 2 moderate white matter disease, multiple mixed (deep more than cortical) cerebral microbleeds, and chronic right basal ganglia IPH, as well as chronic lacunar right paramedian pontine and corona radiata infarcts, pointed to chronic hypertensive vasculopathy as the most likely etiology. Presence of concentric left ventricular hypertrophy on echocardiogram and positive Sokolov criteria on electrocardiogram also corroborated the history of uncontrolled hypertension (Figure 2a–f). Against this background, the acute pontine IPH was best explained as an acute amphetamine‐associated hypertensive injury superimposed on chronic hypertensive vasculopathy, with the chronic vasculopathy as the dominant structural contributor and amphetamine the likely acute precipitant via a sympathetically mediated hypertensive surge.

**FIGURE 2:**
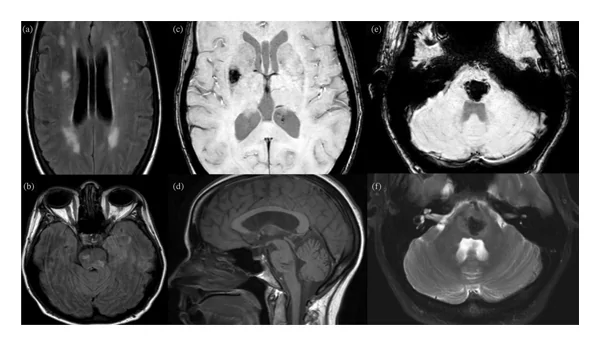
(2a,b) Fluid attenuated inversion recovery (FLAIR) MRI showing Fazekas Grade 2 moderate white matter disease and chronic pontine microvascular changes. (2c,e) Susceptibility‐weighted (SWI) MRI showing acute dorsomedial pons IPH and chronic right lentiform IPH. (2d) Sagittal T1 sequence showing isointense dorsal pontine lesion. (2f) Redemonstrated T2 hypointense lesion in pons.

We performed further ancillary testing to aid hearing loss assessment. Brainstem auditory evoked potentials (BAEPs) were performed using rarefaction polarity click stimuli at 95 dB with a repetition rate of 11.1 Hz and a stimulus frequency of 1 kHz. Absolute latencies and interpeak latencies were preserved bilaterally. There was no electrophysiologic evidence of a demyelinating lesion affecting the brainstem auditory sensory pathway on either side to explain the hearing loss. Additional workup, such as lumbar puncture to rule out infections and otoacoustic emissions (OAEs) to identify cochlear causes of hearing loss, was not necessary as first‐line testing revealed the appropriate diagnosis.

At the 3‐month clinic follow‐up, the patient reported complete subjective resolution of her hearing loss, which was corroborated on neurologic examination by an intact bilateral whisper test. Although she reported mild, nondisabling residual sensorimotor symptoms, she had been able to successfully return to work.

## 3. Discussion

This case emphasizes the importance of treating bilateral SSNHL as a neurologic emergency. We discuss important differentials of SSNHL and the importance of a good neurologic exam in lesion localization.

### 3.1. Bilateral Hearing Loss as a Neurologic Emergency

Bilateral SSNHL is not commonly encountered by neurologists, hence leading to misdiagnosis and delayed treatment. The broad categories and associated underlying etiologies of SSNHL are detailed in Table 1 [3]. Among all these diagnoses, acute ischemic and hemorrhagic strokes should always be ruled out first because of the sudden‐onset nature of the disease, as it may represent an underlying basilar occlusion or hemorrhage, both of which have high morbidity and mortality if not treated promptly [6]. In a recent case series of basilar artery occlusion, sudden bilateral hearing loss and tinnitus were the only presenting symptoms for up to 10 days before patients developed other brainstem symptoms [7]. Chen et al. similarly reported posterior circulation infarction presenting with sudden deafness, emphasizing that hearing loss may serve as an early warning sign of vertebrobasilar ischemic stroke in patients with vascular risk factors [8]. The internal auditory artery or labyrinthine artery arises from the anterior inferior cerebellar artery or basilar artery and is the major blood supply to the inner ear with little to no collateral supply [9]. The mean time from symptom onset to hospital arrival was 13 h in a case series, with most cases initially labeled as benign peripheral vertigo by first‐line providers [7]. Patients who present to the emergency room with nonspecific posterior circulation symptoms (e.g., vertigo and dizziness) account for 130,000–220,000 visits annually, and strokes are initially missed in 30% of them [10]. Zhong et al. proposed that moderate‐to‐severe bilateral hearing loss should always prompt vascular imaging and MRI to rule out posterior circulation stroke [11]. A simple and readily available diagnostic test, like a CT scan of the head, can rule out acute hemorrhage, which in this patient’s case was lifesaving. In her 3‐month follow‐up, she was found to have non‐disabling deficits.

**TABLE 1:** Differential diagnosis of sudden bilateral sensorineural hearing loss.

1. Vascular	Ischemic stroke, intracranial hemorrhage, vasculitis
2. Infections	Meningitis, Mumps, CMV
3. Tumor	Vestibular schwannomas, leptomeningeal carcinomatosis, meningioma, other cerebellopontine angle tumors
4. Toxic	Ototoxic medications, chemotherapy, substance use (cocaine, alcohol, heroin, ecstasy, etc.)
5. Autoimmune	Lupus, Kawasaki’s disease, inflammatory bowel disease, Cogan’s syndrome
6. Iatrogenic	Post operative, complication of anesthesia
7. Idiopathic	No cause identified (most common cause of SSNHL)

### 3.2. Lesion Localization and Neuroanatomy

We propose that the location of the hemorrhage in our patient suggests disruption of fibers in the trapezoid body, which is a key decussating auditory tract in the pontine tegmentum, transmitting bilateral auditory signals from the cochlear nuclei to the superior olivary complexes [12]. Injury to this structure can result in bilateral hearing deficits despite intact peripheral hearing mechanisms. Hoshino et al. reported a similar case of dorsomedial pontine hemorrhage with bilateral hearing loss, supporting the trapezoid body hypothesis [13]. Our patient presented with a more complex pontine syndrome (gaze palsy, pronator drift, ataxia, and sensory loss) compared with Hoshino’s patient (dysarthria and right leg paralysis), reflecting broader involvement of the PPRF and adjacent structures.

### 3.3. Role of BAEPs and Other Otologic Tests in Stroke

BAEP waveforms are generated by specific neural structures: Wave I by the distal auditory nerve, Wave II by the cochlear nucleus, Wave III by the superior olivary complex, and Waves IV–V by the lateral lemniscus and inferior colliculus [14]. Demyelinating lesions usually result in prolonged I–V and/or III–V interpeak latency, while Wave I is preserved [14]. Normal latencies in our patient (presented in Figure 3) suggest an absence of a demyelinating lesion, and normal Wave I coupled with normal otoscopic exam makes a peripheral etiology of hearing loss less likely.

**FIGURE 3:**
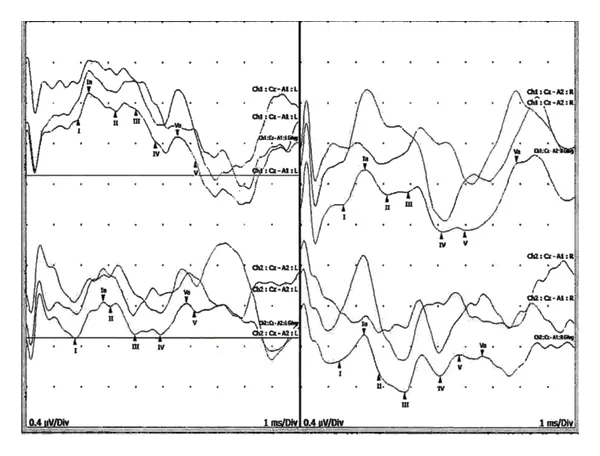
Short latency brainstem auditory evoked potential (BAEP) study of the auditory nerves recording from the bilateral auditory cortex performed using American clinical neurophysiology society standardized montages and protocol shows normal ipsilateral and contralateral absolute latencies for Waves I, II, III, IV, and V, as well as normal ipsilateral and contralateral interpeak latencies between Waves I–III, I–V, and III–V bilaterally. Sensitivity = 0.4 μV/division. Sweep speed = 1 ms/division.

Our patient had normal BAEP waveforms despite bilateral hearing loss—a finding that contrasts with Hoshino et al. [13], who reported poorly defined Wave V bilaterally in a case of dorsomedial pontine hemorrhage. This discrepancy is explained by the anatomical limitations of BAEP testing and suggests BAEPs can have variable results in patients with stroke or structural lesions [15, 16]. Any abnormalities on BAEPs depend on whether the neural generator is significantly affected or whether the large myelinated tracts between the neural generators with the fastest firing action potentials are affected. The trapezoid body is not itself a primary BAEP waveform generator; it is a decussating fiber tract between the cochlear nuclei and superior olivary complex. If a lesion disrupts the decussating fibers while sparing the ipsilateral ascending pathways, BAEP generators can produce normal waveforms. Chu demonstrated that Waves IV and V were abolished only by lesions involving the dorsolateral tegmentum of the upper pons, not by central pontine lesions [17]. Egan et al. and Goyal et al. reported a patient with bilateral total deafness from a midline pontine hemorrhage in whom Waves IV and V were bilaterally preserved [5, 18].

OAEs are another adjunctive test that can be used in the evaluation of SSNHL. OAEs assess cochlear outer hair cell function and can help distinguish sensory (cochlear) from neural (retrocochlear) causes of hearing loss [19]. However, OAEs cannot assess auditory pathways proximal to the cochlea and, therefore, cannot detect central causes of hearing loss. In our case, the sensorineural (central) nature of the deficit was inferred rather than audiometrically proven, on the basis of bedside testing (inability to repeat whispered numbers bilaterally), normal otoscopy excluding an obvious conductive cause, and objective localization of a central lesion by imaging. Formal audiologic testing, including pure‐tone audiometry, speech audiometry, and formal ENT evaluation, was not pursued, which the authors acknowledge as a limitation. Their absence precludes formal audiometric classification and quantification of the hearing loss and means that a cochlear or conductive contribution cannot be entirely excluded. Furthermore, the exact timing and trajectory of hearing recovery could not be determined, as the only documented clinical encounter between admission and follow‐up occurred at 3 months, when complete resolution was reported by the patient.

### 3.4. Clinical Implications

This case reinforces several key clinical messages. First, bilateral SSNHL should be treated as a neurologic emergency and has various etiologies, but ischemic and hemorrhagic stroke should always be ruled out first. Second, a thorough neurologic examination is essential for lesion localization and can distinguish central from peripheral etiologies. Third, normal BAEP does not exclude a central auditory lesion—BAEP is most useful for excluding demyelinating processes and confirming intact peripheral auditory function but has anatomical limitations in detecting structural pontine lesions that spare the neural generators.

## Funding

No funding was received for this manuscript.

## Disclosure

A version of this case was presented as an e‐poster at the annual Society of Vascular and Interventional Neurology meeting in 2025.

## Consent

Written informed consent was obtained from the patient for publication of this case.

## Conflicts of Interest

The authors declare no conflicts of interest.

## Data Availability

The data that support the findings of this study are available on request from the corresponding author. The data are not publicly available due to privacy or ethical restrictions.
